# Effects of the Carbohydrate Sources Nectar, Sucrose and Invert Sugar on Antibacterial Activity of Honey and Bee-Processed Syrups

**DOI:** 10.3390/antibiotics10080985

**Published:** 2021-08-15

**Authors:** Veronika Bugarova, Jana Godocikova, Marcela Bucekova, Robert Brodschneider, Juraj Majtan

**Affiliations:** 1 Laboratory of Apidology and Apitherapy, Department of Microbial Genetics, Institute of Molecular Biology, Slovak Academy of Sciences, Dubravska Cesta 21, 845 51 Bratislava, Slovakia; veronika.bugarova@savba.sk (V.B.); jana.godocikova@savba.sk (J.G.); marcela.bucekova@savba.sk (M.B.); 2 Institute of Biology, University of Graz, Universitätsplatz 2, A-8010 Graz, Austria; robert.brodschneider@uni-graz.at; 3 Department of Microbiology, Faculty of Medicine, Slovak Medical University, Limbova 12, 833 03 Bratislava, Slovakia

**Keywords:** medical-grade honey, bacteria, glucose oxidase, honeybee, functional food

## Abstract

Honey is a functional food with health-promoting properties. Some types of honey are used in wound care for the treatment of acute and chronic infected wounds. Increased interest in using honey as a functional food and as a base for wound care products causes limited availability of raw honey. Numerous studies suggest that the protein content of honey is mainly comprised of bee-derived proteins and peptides, with a pronounced antibacterial effect. Therefore, the aim of our study was to characterize for the first time the antibacterial activity of raw honeys and bee-processed syrups which were made by processing sucrose solution or invert sugar syrup in bee colonies under field conditions. Furthermore, we compared the contents of glucose oxidase (GOX) and the levels of hydrogen peroxide (H_2_O_2_) in honey samples and bee-processed syrups. These parameters were also compared between the processed sucrose solution and the processed invert sugar syrup. Our results clearly show that natural honey samples possess significantly higher antibacterial activity compared to bee-processed syrups. However, no differences in GOX contents and accumulated levels of H_2_O_2_ were found between honeys and bee-processed syrups. Comparison of the same parameters between bee-processed feeds based on the two artificial carbohydrate sources revealed no differences in all measured parameters, except for the content of GOX. The amount of GOX was significantly higher in bee-processed sucrose solutions, suggesting that processor bees can secrete a higher portion of carbohydrate metabolism enzymes. Determination of honey color intensity showed that in bee colonies, bee-processed syrups were partially mixed with natural honey. Further research is needed to identify the key botanical compounds in honey responsible for the increased antibacterial potential of honey.

## 1. Introduction

Honey is a natural product and functional food with proven therapeutic advantages in the treatment of various disorders [1]. Besides its oral consumption, honey has successfully been used topically in wound care for a broad spectrum of injuries and burns [2]. Its biological properties, including antibacterial, anti-biofilm, anti-inflammatory, and regenerative activities are mandatory characteristics which may vary from honey to honey or might be significantly affected by both environmental conditions and technological processing. In this sense, the determination of all mentioned biological properties or, at least, honey antibacterial activities, is crucial to obtain honey for medicinal purposes. Currently, there are no quality parameters or regulations for raw honey registration as a medical-grade honey or its uses as a major ingredient of medical-grade products [3]. However, medical-grade honey should be free of contaminants, sterile, and safe and must meet all criteria adopted for medical devices. In addition, honey has frequently been impregnated with other materials (e.g., gelatine, alginate, agarose) and can be used in the form of gels, dressings or ointments.

Numerous commercial honey-based medicinal formulations recommended for the treatment of different disorders have exhaustedly been listed in a recent review [4]. The market of honey-based formulations grows continuously and, thus, a high demand for honey sources globally can be expected. Likewise, there is increased interest in using honey as a functional food, and the availability of natural honey might become limited. Therefore, new attempts to prepare medical-grade honeys and honey-based formulations are needed.

Recently, a new bioengineered honey (SurgihoneyRO^TM^) has been developed and validated for use in wound care. This synthetic medical product comprises gamma-irradiated unspecified honey and artificially added glucose oxidase (GOX) from *Aspergillus niger* [5]. Furthermore, a novel synthetic product (Synthetic RO+) which mimics the honey’s carbohydrate composition and contains fungal GOX has recently been developed as an alternative option to honey-based medical products [6]. Both these medical products are solely based on the bactericidal activity of continuously generated hydrogen peroxide (H_2_O_2_), mediated by fungal GOX in the presence of glucose and water. The GOX found in natural honey is a regular but quantitatively variable bee-derived component of each type of natural honey [7]. The levels of H_2_O_2_ may differ from honey to honey, and several factors may affect the total concentration of H_2_O_2_ [8]. Although H_2_O_2_ is considered to be a key antibacterial compound in diluted honey, some studies have shown that its level in different honeys does not correlate with the overall antibacterial activity [8,9,10]. Taken together, it is questioned whether synthetic honey-like products based solely on the action of accumulated H_2_O_2_ are more effective in bacterial growth inhibition than natural honeys. Moreover, phytochemicals, including polyphenols/flavonoids found in honey, can increase the antibacterial activity of natural honey via synergy with other biologically active components [10]. Last but not least, honey promotes and speeds up wound healing through several distinct mechanisms, where different honey molecules are involved in the healing process [2].

Honey bee colony health and survival are intensively investigated, as high colony losses have been reported from many countries, resulting in high economic costs [11,12,13,14,15]. Next to the well-established pests and pathogens as well as environmental factors impeding honey bee health, the quality of winter feed provided as substitute to honeybees is also important. Several studies have been conducted to identify the best possible carbohydrate feed for honey bees on different levels, in the laboratory and in the field [16,17,18,19,20]. Bees consume the sucrose or invert sugar syrups (constituted mostly of fructose and glucose) and store them after a process similar to the production of honey in cells that are finally sealed. Bees probably add enzymes during this process, but in contrast to the physicochemical properties of syrups processed and stored by bees [21], the antibacterial activity, levels of hydrogen peroxide and GOX have not been studied.

The aim of this study was to characterize the antibacterial potential of Austrian natural honeys for the first time and to compare its overall antibacterial efficacy to commercially available manuka honey. Available bee colonies were fed with either sucrose or invert sugar syrup (an equimolar mixture of glucose and fructose), and the overall antibacterial effect of raw honeys and bee-processed syrups was compared within particular bee colonies to eliminate environmental and genetic differences. Furthermore, the content of GOX and the level of H_2_O_2_ were determined in natural honeys and bee-processed syrups. Finally, we compared the antibacterial activity and content of GOX between sucrose and inverted sugar syrups after their processing by bees. Our results can facilitate the understanding of the effects of various food diets on wintering honey bees, such as honey, as in modern beekeeping, bee-processed food from different carbohydrates is the only energy source for colonies in winter [22,23].

## 2. Results

### 2.1. Antibacterial Activity of Styrian Honey Samples

**Figure 1:**
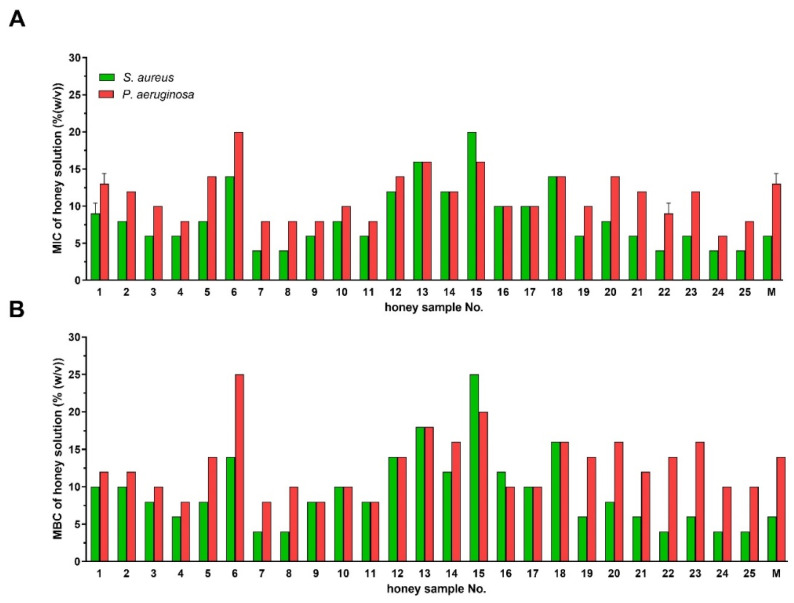
Antibacterial activity of Styrian honey samples (*n* = 25) and manuka honey UMF 15+ (M) against *Staphylococcus aureus* and *Pseudomonas aeruginosa* isolates. Activity was determined with a minimum inhibitory concentration (MIC) (**A**) and a minimum bactericidal concentration (MBC) assay (**B**). MIC and MBC were defined as the lowest concentrations of honey solution (%) inhibiting bacterial growth and killing the bacteria, respectively. Data are expressed as the mean values with SD.

The antibacterial activity of the tested honey samples was expressed as MIC/MBC of honey samples (Figure 1). The lowest MIC value determined in honey samples was 4%. The MBC values were either identical or slightly higher compared to the MIC values. The highest MIC and MBC values of honey samples were 20 and 25%, respectively. The average value of MIC of manuka honey were 6 and 13% against *S. aureus* and *P. aeruginosa*, respectively. Honey samples were more effective against *S. aureus* than *P. aeruginosa*. None of the tested honey samples achieved the poor antibacterial activity of artificial honey, which was 45% (sugar solutions only).

### 2.2. Comparison of Antibacterial Activities of Natural Honeys and Bee-Processed Syrups

**Figure 2:**
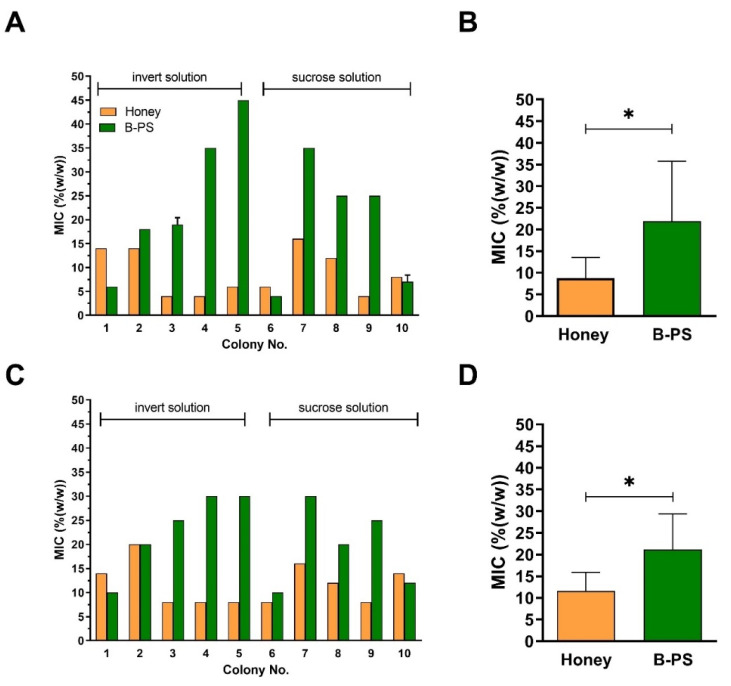
Antibacterial activities of raw honey samples and bee-processed syrups (B-PS) from bee colonies (*n* = 10) against *Staphylococcus aureus* (**A**,**B**) and *Pseudomonas aeruginosa* (**C**,**D**) isolates. Honey and bee-processed syrups were subsequently collected from the same colonies. Activity was determined with a minimum inhibitory concentration (MIC) assay. (**B**,**D**) Average MIC values for honey samples and bee-processed syrups. Data are expressed as the mean values with SD. * *p* < 0.05 (Wilcoxon test).

To characterize the antibacterial potential of different winter feeds (sucrose and invert sugar syrup) after their uptake and processing in the colonies, we determined the antibacterial activity of honey samples (*n* = 10) and compared them to that of bee-processed syrups (*n* = 10; where *n* = 5, sucrose solution and *n* = 5, invert sugar syrups) within particular bee colonies, i.e., compared the activity of honey harvested before feeding and the syrups stored by bees fed after honey. Figure 2 shows the antibacterial activities of honey samples with their paired bee-processed syrups. Honey samples exhibited higher antibacterial activity compared to bee-processed syrups, except for samples No. 1, 6, and 10. The mean of antibacterial activity expressed as the MIC value of honey samples against *S. aureus* and *P. aeruginosa* was 8.8 and 11.6%, respectively (Figure 2B,D). Overall, the antibacterial activity of bee-processed syrups was significantly lower (*p* = 0.037 for *Staphylococcus aureus*, *p* = 0.031 for *Pseudomonas aeruginosa*, Wilcoxon test) compared to that of honey samples against both tested bacterial species (Figure 2B,D).

### 2.3. Content of GOX and Generation of H_2_O_2_ in Honeys and Bee-Processed Syrups

**Figure 3:**
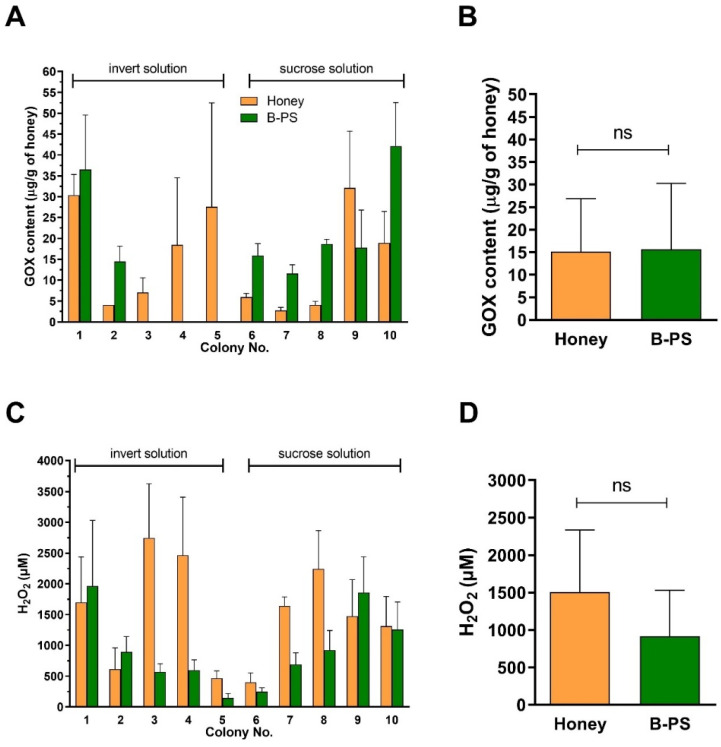
GOX contents and H_2_O_2_ levels in raw honey samples and bee-processed syrups (B-PS) from bee colonies (*n* = 10) (**A**,**C**). Honey and bee-processed syrup were subsequently collected from the same colonies. (**B**,**D**) Average GOX content and levels of H_2_O_2_ for honey samples and bee-processed syrups. Data are expressed as the mean values with SD.

The antibacterial properties of honey largely depend on the accumulation of H_2_O_2_, which is mainly generated by the enzymatic conversion of glucose catalyzed by GOX enzyme present in all types of honey. In this study, GOX was immunodetected and quantified in all raw honeys and most of the bee-processed syrup samples. The amounts of GOX and H_2_O_2_ in honey and bee-processed syrup samples are shown in Figure 3. Although the average amounts of GOX quantified in honey and bee-processed syrups were almost identical (*p* = 0.915, t-test) (15.1 µg/g in honey vs. 15.7 µg/g in bee-processed syrups), we did not detect GOX in three bee-processed syrup samples (No. 3, 4 and 5) (Figure 3A). The average concentrations of H_2_O_2_ in honey and paired bee-processed syrup were 1507 ± 830 µM and 915 ± 616 µM, respectively (Figure 3D). However, the H_2_O_2_ levels in bee-processed syrup and honey were not statistically significant (*p* = 0.076, t-test).

**Figure 4:**
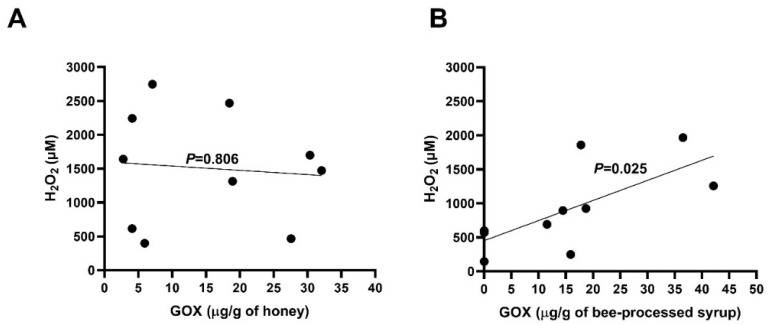
Correlation between the content of GOX and H_2_O_2_ production capacity in (**A**) raw honey samples and (**B**) bee-processed syrups. A Pearson correlation test was used for analysis.

No correlation (*p* = 0.806, Pearson test) between the GOX amount and H_2_O_2_ level in honey samples was found (Figure 4A). On the other hand, a significant correlation (*p* = 0.025, Pearson test) between the same parameters in bee-processed syrups was observed (Figure 4B).

### 2.4. Comparison of Antibacterial Activity and Contents of GOX and Proteins between Sucrose and Inverted Sugar Feeds after Their Processing by Honey Bees

**Figure 5:**
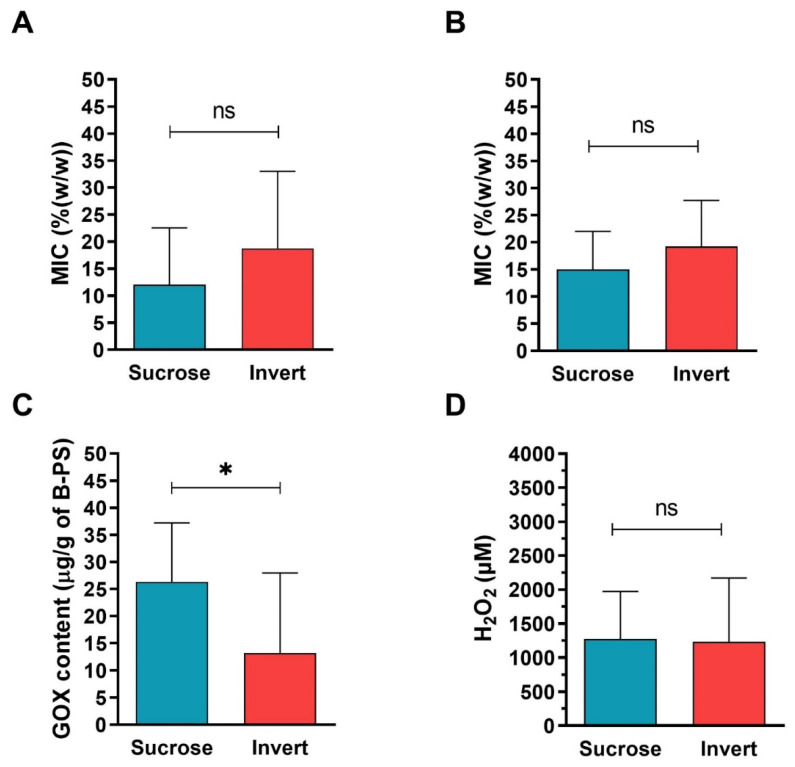
Antibacterial activity of bee-processed syrups (B-PS) and GOX contents and H_2_O_2_ levels in B-PS collected after processing sucrose (*n* = 11) or invert sugar (*n* = 8) feeds in the bee colony. Average antibacterial activities of B-PS against Staphylococcus aureus (**A**) and Pseudomonas aeruginosa (**B**). Average GOX contents (**C**) and levels of H_2_O_2_ (**D**) in BP-S. Data are expressed as the mean values with SD. * *p* < 0.05 (unpaired *t*-test).

The antibacterial activity of all collected bee-processed syrups, including sucrose (*n* = 11) and inverted sugar (*n* = 8) processed syrups from different bee colonies, was determined and expressed as the MIC value. No significant difference in antibacterial activity between the bee-processed sucrose and inverted sugar syrup samples was found. The MIC values of all bee-processed syrups (*n* = 19) ranged from 6 to 45% and 8 to 30% against *S. aureus* and *P. aeruginosa*, respectively (Figure 5). Bee-processed sucrose syrup exhibited slightly higher antibacterial activity against both bacteria compared to processed invert sugar feeds, although this difference was not statistically significant (*p* = 0.183, Mann Whitney test, *Staphylococcus aureus*; *p* = 0.298, Mann Whitney test, *Pseudomonas aeruginosa*) (Figure 5A,B). The antibacterial activity of bee-processed sucrose syrup is due to the statistically significant increased (*p* = 0.039, unpaired t-test) average level of GOX in these samples in comparison to bee-processed invert syrups (Figure 5C). Representative images of western blot analysis of GOX in bee-processed syrups are shown in Figure S1. On the other hand, the average levels of H_2_O_2_ were similar in both groups of bee-processed syrup (*p* = 0.912, unpaired t-test) (Figure 5D).

**Figure 6:**
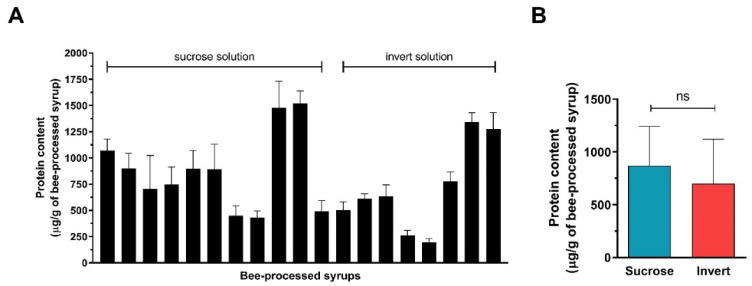
Total protein contents of bee-processed syrups. Protein content was determined using the Quick Start Bradford protein assay. (**A**) Protein content in individual bee-processed syrup after processing sucrose solution (*n* = 11) or invert sugar syrup (*n* = 8). (**B**) Average protein content in both analyzed groups of bee-processed syrups.

The total protein content was determined in bee-processed sucrose and invert sugar syrups (Figure 6). The average protein content in bee-processed sucrose feed and invert sugar feed was 870 ± 373 and 700 ± 421 µg/g, respectively. No significant difference (*p* = 0.363, unpaired t-test) in the protein content of the bee-processed sucrose and inverted sugar feed samples was found.

### 2.5. Colour Analysis of Honey and Bee-Processed Syrups

**Figure 7:**
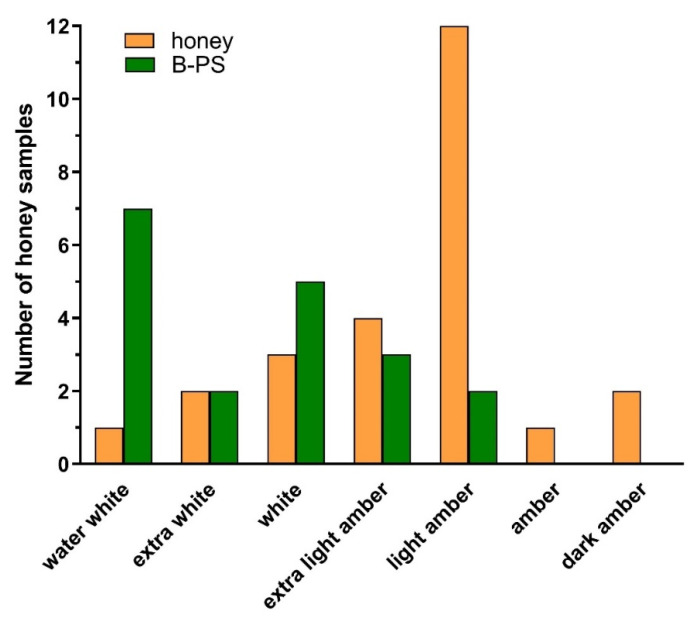
Color analysis of honey samples and bee-processed syrups. The color-grading system consisted of seven different colors (water white, extra white, white, extra light amber, light amber, amber and dark amber).

Most honey samples (76%) showed a darker (amber) color, indicating the presence of phytochemicals responsible for the coloring of honey (Figure 7). On the other hand, the bee-processed syrup samples were lighter in color. However, some bee-processed syrups showed lighter amber color, suggesting their mixture with pure honey.

## 3. Discussion

The antibacterial activity of honey is one of the most studied biological properties of honey and is essential for medical-grade honey. However, the qualitative parameters of medical-grade honey have not yet been defined. Several studies have provided compelling evidence that every raw honey sample regardless of its botanical and geographical origin exhibits antibacterial activity, irrespective of the sugar content alone [7,24,25,26]. Identification of key antibacterial compounds, namely GOX and methylglyoxal (MGO), responsible for the pronounced antibacterial activity of different honeys, opens a new avenue in the fabrication of engineered artificial honey-like wound care products. These honey-like wound care products have several advantages such as precise and reproducible composition, the absence of environmental contaminants, and independence on beekeeping. On the other hand, honey, as a natural product, contains more than 200 different compounds with various pharmacological effects, which can act in synergy with each other to augment the overall antibacterial effect of honey. We have recently shown that vitamin C, commonly found in honey, significantly increased honey antibacterial activity against bacterial pathogens and supported the eradication of multi-bacterial biofilms [27]. Furthermore, bee-derived GOX enzyme may possess a higher stability, specificity and enhanced activity than fungal GOX enzyme which is used as a biologically active component of engineered bee-processed syrups.

In the present study, we characterized the effects of different carbohydrate sources (nectar, sucrose, and invert syrup) on the antibacterial activity of honey and bee-processed syrups. In 10 bee colonies, we compared the overall antibacterial efficacy, the GOX content, and the level of H_2_O_2_ between natural raw honey samples and processed feeding samples (sucrose or invert syrup) in the same colony. Furthermore, we also analyzed the difference in measured activity and parameters between processed sucrose and invert syrups.

The antibacterial efficacy of natural honey samples against both tested bacteria was significantly higher when compared to that of bee-processed syrups. Interestingly, the GOX content and the level of H_2_O_2_ did not differ between honey samples and bee-processed syrups, suggesting that bees add enzymes during the processing of the syrup similar to those when processing honey. The difference in the antibacterial activities of bee-processed syrups and natural honey must therefore be derived from other compounds of botanical origin. Furthermore, a significant correlation between the concentration of GOX and the level of H_2_O_2_ was found in bee-processed syrups but not in in raw honey samples. Similarly, previous studies did not show any correlation between the GOX content and the generated H_2_O_2_ in blossom and honeydew honey samples [7,24]. The presence of catalase which neutralizes H_2_O_2_, is one of the major factors responsible for these observations. The concentration of catalase in bee-processed syrups should be negligible because of its bacterial/plant origin.

Glucose oxidase is expressed in the hypopharyngeal glands of foragers [28], nurses and winter worker bees [29,30] as well as food storer bees [31]. Recently, Lewkowski and co-workers [32] analyzed the protein content in honey-like products generated from different feeding regimes (sucrose and invert syrup). Mass spectrometric analyses of honey-like products revealed that major royal jelly proteins (MRJPs), alpha-glucosidase and GOX were the most dominant proteins [32]. No difference in protein quality and quantity was found among the two studied honey-like products. These findings are partially in agreement with our results where no significant difference in the protein content was found between the group of processed sucrose products and that of processed invert syrup. On the other hand, we found a significant difference in the GOX content between bee-processed sucrose and invert sugar syrup. One of the possible explanations is that bees can respond behaviorally and electrophysiologically to different kinds of sugar such as sucrose, fructose, maltose, and glucose [33] and thus may secrete a higher portion of carbohydrate metabolism enzymes from hypopharyngeal glands to the sucrose solution than invert syrup in order to digest the sucrose to glucose and fructose. For invertase, the correlation of enzyme secretion during the ripening process of honey and sucrose solutions has been demonstrated [34]. The release of different enzymes by bees may be coupled, so that our findings and those of Lichtenberg-Kraag support the assumption that sucrose-rich diets constitute a higher enzymatic effort for bees compared to invert sugar syrups. This interesting hypothesis warrants further research, including examinations of the content and activity of secreted bee-derived enzymes.

From the beekeeping point of view, it is common practice to use sucrose and invert syrups for the feeding of bees [17]. Winter feeding with commercially available syrups is an economically feasible alternative to costly wintering on honey for most conventional beekeepers. The quality of food for overwintering success and bee fitness is essential; it also impacts resistance to diseases [35]. According to the results of a two-year field trial, the best fitness parameters and the lowest prevalence and intensity of nosematosis, a common bee disease, were found in colonies fed with honey compared to other substances (sucrose, inverted sugar and wheat starch syrup) [35]. Honey provides reasonable protection from bacterial infections and, as shown in this study, possesses significantly higher antibacterial potential than supplemental syrups after their processing in the colony. Commercially available inverted sugar and starch syrups may contain high levels of the toxic hydroxymethylfurfural (HMF), which negatively affects bee health. The HMF content rises sharply in syrups during long-term storage [21,36,37].

Many studies investigating different syrups in laboratory settings found differences between honey and different artificial carbohydrate sources, including between sucrose and invert or high-fructose corn syrups (HFCS). Hundreds of differences in gene expression in the fat body of bees fed sucrose solution or HFCS relative to honey are known [18]. The beneficial physiological functions of honey have often been attributed to plant phytochemicals that up-regulate detoxification or immunity genes [38,39,40]. Our results on the presence of GOX and the low but proven antibacterial activity of bee-processed syrups also have implications for future investigations. Instead of feeding the raw (unprocessed) syrups to caged bees, investigations of feeding syrups after processing by bees in a hive are more realistic to understand honeybee nutrition and optimal carbohydrates for bee health.

Bee-processed syrups were collected from bee colonies under field conditions, and forager bees were not precluded from collecting nectar and pollen. Our results of the color analysis suggest that bee-processed syrups were partially mixed with raw natural honey. The ripening process of nectar/syrup is a complex process including the repeated relocation of the contents of many storage cells and many bee contacts before the final product is stored in cells sealed with a wax layer. Eyer et al. showed that honey is an inhomogeneous matrix due to the relocation of the content of many cells in honeycomb before final storage as a part of the ripening process in bee hive [41]. We therefore assume that plant compounds, including polyphenols and flavonoids, can be partially responsible for the antibacterial activity of those bee-processed syrups which were mixed with natural honey directly in the beehive.

Adulteration of honey is a known problem, and sophisticated analytical methods for honey authenticity and the detection of foreign sugars in honey have been developed [42]. The antibacterial activity of honey is an important property of honey, but it has not yet been included in international honey standards [43]. Our results indicate a lower but present antibacterial activity in bee-processed syrups. These bee-processed syrups clearly cannot be defined as honey [44]. Antibacterial activity assays alone are therefore not a decisive criterion in detecting honey fraud, but they should be used in combination with other analyses.

## 4. Materials and Methods

### 4.1. Sampling of Honeys and Bee-Processed Syrups

**Figure 8:**
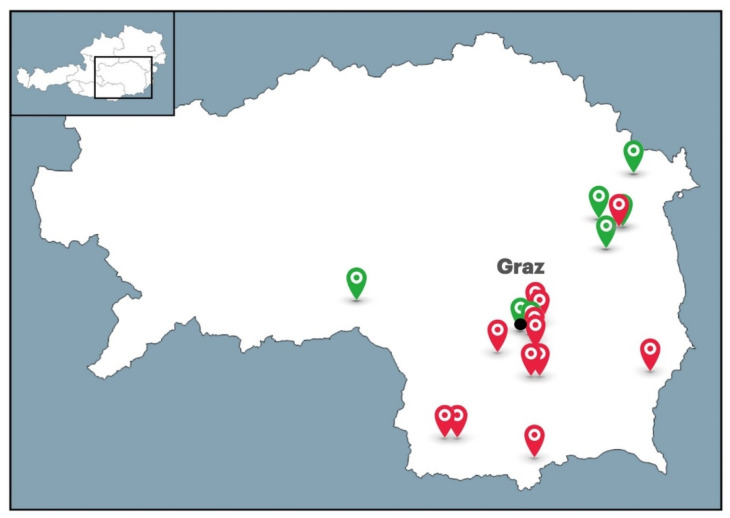
Honey sampling locations in Styria (Austria). Honey samples (*n* = 25) were collected in the year 2020. Red color–honey samples; green color–honey samples and paired bee-processed syrups.

Raw honey samples (*n* = 25) were collected from beekeepers from different parts of Styria (Austria) in July/August 2020 (Figure 8). The honey samples included only liquid honeys from mostly polyfloral sources (Table 1). Honey samples were extracted either from one colony (sealed honey comb was sampled) or multiple colonies (honey extracted from more than one colony belonging to one apiary). In late August and early September, bee-processed syrups (*n* = 19) were collected from sealed cells after feeding the bees either by sucrose solution (*n* = 11) or inverted sugar syrup (*n* = 8). Overall, 10 colonies were available and used to study the antibacterial efficacy of raw honey samples and bee-processed syrups collected from the same colony. After honey harvesting, bees were fed either with sucrose solution or invert sugar syrup. Honeys and bee-processed syrups were collected into sterile plastic containers, and all samples were immediately stored at 4 °C in the dark. Commercially available manuka honey UMF 15+ (Comvita, New Zealand) was purchased from a local shop.

### 4.2. Microorganisms

The antibacterial activity of the honey samples was assessed against *Pseudomonas aeruginosa* CCM1960 and *Staphylococcus aureus* CCM4223 isolates, obtained from the Department of Medical Microbiology, Slovak Medical University in Bratislava, Slovakia.

### 4.3. Honey Antibacterial Inhibition Assays

The minimal inhibitory concentration (MIC) and minimal bactericidal concentration (MBC) of honey and bee-processed syrup samples were determined using *S. aureus* and *P. aeruginosa*, following the method of Bucekova et al. [7]. Bacteria were cultured overnight in Mueller-Hinton broth (MHB) at 37 °C and subsequently suspended in phosphate-buffered saline (PBS), pH 7.2; the turbidity of the suspension was adjusted to 10^8^ colony-forming units (CFU)/mL, and the suspension was diluted with MHB medium to a final concentration of 10^6^ CFU/mL. Subsequently, 10 μL of the diluted bacterial suspension were inoculated into sterile 96-well polystyrene U-shape plates (Sarstedt, Nümbrecht, Germany), where each well contained 90 μL of diluted honey and sugar solution samples of different concentrations so the final volume in each well was 100 μL. We incubated 96-well plates containing mixtures of diluted honey or sugar solutions with bacterial suspensions for 18 h at 37 °C, 1250 rpm, and the inhibition of bacterial growth was determined visually. The MIC values represented the lowest possible concentration of honey or feed sample at which bacterial growth was inhibited. All tests were performed in triplicate and repeated three times. Each honey sample dilution was prepared from a 50% honey solution (*w*/*w* in MHB medium) by further dilution with the MHB medium, resulting in final concentrations of 40, 35, 30, 25, 20, 18, 16, 14, 12, 10, 8, 6, and 4%.

The minimum bactericidal concentration (MBC) of honey and bee-processed syrup samples was also evaluated. The viability of bacteria in wells with no turbidity (growth) was determined by dropping 2 μL of each well onto an MHB agar plate and incubating at 37 °C for 24 h. The lowest concentration of honey and bee-processed syrup that did not show bacterial growth was considered as MBC.

### 4.4. Determination of the GOX Content

The GOX content was determined semi-quantitatively according to Bucekova et al. (2019) [7]. Briefly, aliquots (15 μL) of 50% (*w*/*w*) honey solution were resolved by SDS-PAGE and the proteins were transferred onto a 0.22-μm nitrocellulose Advantec membrane (Sigma-Aldrich, Germany) using the wet blotting procedure. The membrane was blocked for 1 h in a Tris-buffered saline-Tween (TBST) buffer (50 mM Tris-HCl, pH 7.5, 200 mM NaCl and 0.05% Tween 20) containing 5% non-fat dried milk and incubated overnight with a rabbit polyclonal antibody against honey bee GOX (Clone No. RB3232; 1:2000 in TBST), which had been prepared by GenCust Europe (Dudelnag, Luxembourg). After washing with TBST, the membranes were incubated for 2 h in blocking buffer containing goat anti-rabbit horseradish peroxidase-linked antibodies (1:2500 in TBST; Promega, USA). Immunoreactive bands were detected in a solution containing dissolved SigmaFast 3,3-diaminobenzidine tablets (Sigma-Aldrich), and specific bands were quantified by densitometry using the ImageJ software (NIH, USA).

Total proteins in bee-processed syrups were measured using the Quick Start Bradford protein assay (Bio-Rad, USA) as described in the instruction manual.

### 4.5. Determination of H_2_O_2_ Concentration

The H_2_O_2_ concentration in honey and bee-processed syrup samples was determined with a Megazyme GOX assay kit (Megazyme International Ireland Ltd.), which is based on H_2_O_2_ release. As a standard, H_2_O_2_ diluted to 9.8–312.5 μM was used. Briefly, 40% (*w*/*w*) honey and bee-processed syrup solutions in 0.1 M potassium phosphate buffer (pH 7.0) were prepared and either immediately used for H_2_O_2_ measurement or measured after 24 h incubation of the prepared solutions at 37 °C. Each honey, bee-processed syrup sample and standard was tested in duplicate in a 96-well microplate and absorbance was measured at 510 nm using a Synergy HT microplate reader (BioTek Instruments, Winooski, VT, USA).

### 4.6. Determination of Colour according to the Pfund Scale

Honey samples and bee-processed syrups (50% aqueous solution, *w*/*v*) were heated to 50 °C to dissolve sugar crystals and the color was determined spectrophotometrically at λ = 635 nm. The honeys were classified according to the Pfund scale after conversion of the absorbance values (Abs) [45]: Pfund (mm) = 38.70 + 371.39 *Abs

The U.S. Department of Agriculture has classified honey into seven color categories: water white, extra white, white, extra light amber, light amber, amber, and dark amber. The Pfund color scale may provide an accurate, inexpensive and convenient methodology for measuring the color intensity of honey as a distance (in mm) in the chromatic space. The respective values (in mm) are as follows: <9 for water white, 9–17 for extra white, 18–34 for white, 35–50 for extra light amber, light amber 51–85, amber 86–114 and dark amber > 114 (USDA Agricultural Marketing Service, 1985).

### 4.7. Statistical Analysis

The Shapiro-Wilk test of normality was used to determine the data distribution. The Wilcoxon test or the t-test for paired honey and bee-processed syrup were used depending on the calculated normality. The Mann Whitney test and the t-test for non-paired sucrose and invert sugar bee-processed syrup samples were used depending on the calculated normality. Data with *p*-values smaller than 0.05 were considered statistically significant. All statistical analyses were performed using GraphPad Prism (GraphPad Software Inc., La Jolla, CA, USA).

## 5. Conclusions

In conclusion, our present study shows that the antibacterial activity of natural honeys exhibited a significantly higher antibacterial activity compared to bee-processed sucrose solution and invert sugar syrup. However, no difference in the GOX content and the level of H_2_O_2_ was found between natural honeys and bee-processed syrups. Comparison of the same parameters between both groups of processed feeds revealed no differences in all measured parameters, except for the content of GOX. The amount of GOX was significantly higher in processed sucrose solutions, suggesting the ability of processor bees to secrete a higher portion of carbohydrate metabolism enzymes to invert (or digest) the sucrose. This suggests a sensual perception of the disaccharide and physiological reaction by honeybees, a hypothesis warranting further research.

## Figures and Tables

**Table 1:** Austrian honey samples analyzed in study.

Honey Sample	Botanical Origin	Sample Extraction from Colony *
1	polyfloral	>1
2	chestnut	>1
3	linden	>1
4	linden	1
5	linden	1
6	polyfloral	1
7	polyfloral	1
8	polyfloral	1
9	linden	1
10	polyfloral	>1
11	linden	1
12	polyfloral	1
13	polyfloral	1
14	polyfloral	1
15	polyfloral	1
16	polyfloral	1
17	polyfloral	1
18	linden	1
19	polyfloral	>1
20	chestnut	>1
21	linden	>1
22	linden/honeydew	>1
23	chestnut	>1
24	polyfloral	>1
25	linden	1

* Honey was extracted either from one colony or multiple colonies.

## Data Availability

The data presented in this study are available on request from the corresponding author.

## Supplementary Materials

The following are available online at https://www.mdpi.com/article/10.3390/antibiotics10080985/s1, Figure S1: Representative western blots of GOX detection in bee-processed sucrose (Su) and invert sugar (In) syrups, The GOX content in bee-processed syrups was determined semi-quantitatively according to Bucekova et al. (2019).
