# Supplementary material for: Effects of the Carbohydrate Sources Nectar, Sucrose and Invert Sugar on Antibacterial Activity of Honey and Bee-Processed Syrups
Source: Antibiotics (Basel). 2021 Aug 15;10(8):985. doi: 10.3390/antibiotics10080985 (PMC8388957; doi:10.3390/antibiotics10080985)
Supplement: Supplementary file 1 [file antibiotics-10-00985-s001.zip › antibiotics-1330202-supplementary.pdf]

antibiotics

MDPI

Article

Effects of the carbohydrate sources nectar, sucrose and invert sugar on antibacterial activity of honey and bee-processed syrups

Veronika Bugarova [1], Jana Godocikova [1], Marcela Bucekova [1], Robert Brodschneider [2] and Juraj Majtan [1,3,*]

[1] Laboratory of Apidology and Apitherapy, Department of Microbial Genetics, Institute of Molecular Biology, Slovak Academy of Sciences, Dubravska cesta 21, 845 51 Bratislava, Slovakia; veronika.bugarova@savba.sk (V.B.), jana.godocikova@savba.sk (J.G.), marcela.bucekova@savba.sk (M.B.), juraj.majtan@savba.sk (J.M.)

[2] University of Graz, Institute of Biology, Universitätsplatz 2, A-8010 Graz, Austria; robert.brodschneider@uni-graz.at (R.B.)

[3] Department of Microbiology, Faculty of Medicine, Slovak Medical University, Limbova 12, 833 03 Bratislava, Slovakia; juraj.majtan@savba.sk (J.M.)

* Correspondence: juraj.majtan@savba.sk; Tel.: +421 903 869413

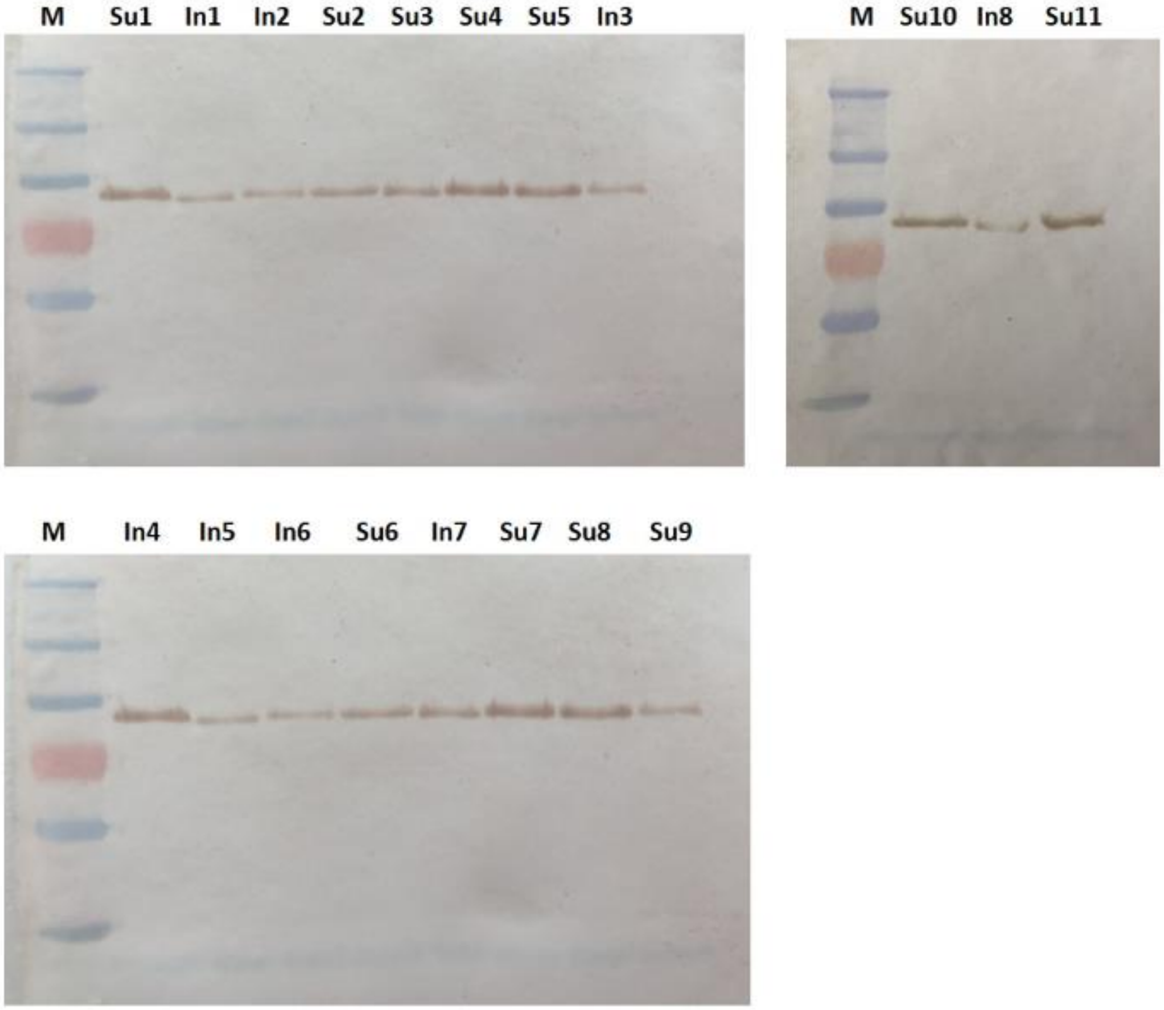

Figure S1. Representative western blots of GOX detection in bee-processed sucrose (Su) and invert sugar (In) syrups, The GOX content in bee-processed syrups was determined semi-quantitatively according to Bucekova et al. (2019) [1].

References:

1.Bucekova, M.; Jardekova, L.; Juricova, V.; Bugarova, V.; Di Marco, G.; Gismondi, A.; Leonardi, D.; Farkasovska, J.; Godocikova, J.; Laho, M.; Klaudiny, J.; Majtan, V.; Canini, A.; Majtan, J., Antibacterial activity of different blossom honeys: new findings. Molecules 2019, 24, E1573.
